# Clusterin Expression in Colorectal Carcinomas

**DOI:** 10.3390/ijms241914641

**Published:** 2023-09-27

**Authors:** Teresa Téllez, Desirée Martin-García, Maximino Redondo, Marilina García-Aranda

**Affiliations:** 1 Surgical Specialties, Biochemistry and Immunology Department, Faculty of Medicine, University of Málaga, 29010 Malaga, Spain; teresatellez@uma.es (T.T.); desirermg@uma.es (D.M.-G.); 2 Red de Investigación en Servicios de Salud en Enfermedades Crónicas (REDISSEC), Red de Investigación en Cronicidad, Atención Primaria y Promoción de la Salud (RICAPPS), Instituto de Investigación Biomédica de Málaga (IBIMA), 29590 Malaga, Spain; marilina.garcia.sspa@juntadeandalucia.es; 3 Instituto de Investigación Biomédica de Málaga y Plataforma en Nanomedicina—IBIMA Plataforma BIONAND, 29590 Malaga, Spain; 4 Research and Innovation Unit, Hospital Costa del Sol, 29602 Marbella, Spain

**Keywords:** clusterin, colorectal cancer, biomarker, tumorigenesis, targeted treatment, personalized medicine

## Abstract

Colorectal cancer is the third most diagnosed cancer, behind only breast and lung cancer. In terms of overall mortality, it ranks second due to, among other factors, problems with screening programs, which means that one of the factors that directly impacts survival and treatment success is early detection of the disease. Clusterin (CLU) is a molecular chaperone that has been linked to tumorigenesis, cancer progression and resistance to anticancer treatments, which has made it a promising drug target. However, it is still necessary to continue this line of research and to adjust the situations in which its use is more favorable. The aim of this paper is to review the current genetic knowledge on the role of CLU in tumorigenesis and cancer progression in general, and discuss its possible use as a therapeutic target in colorectal cancer.

## 1. Introduction

Clusterin (CLU), also known as lipid-binding protein type A2 or Apolipoprotein J (ApoJ), SPG2, TRPM-2 or CLU is a molecular chaperone that was first discovered in 1979 in rat testis, where it was observed to cause cell aggregation in vitro [1]. This protein is present in human tissues and body fluids, such as blood, saliva, cerebrospinal fluid, and synovial fluid, which have an important role in processes such as cell adhesion, immune response, and cell survival [2,3,4]. CLU has also been shown to inhibit protein aggregation under cellular stress by binding to misfolded proteins, resulting in the accumulation of inactive but stabilized proteins, which can regain their functionality with the help of other folding chaperones [5]. In stressful cellular situations, such as exposure to chemotherapeutic drugs, hypoxia, or ionizing radiation, increase clusterin levels as part of their defense mechanism [6].

The human *CLU* gene, located on chromosome 8p21-p12, consists of 11 exons and approximately 16 kilobases. *CLU* produces three main transcription products [7], Variant 1: NM_001831, Variant 2: NR_038335 and Variant 3: NR_045494, that share exons 2–11 but differ in exon 1 (1a, 1b, 1c), suggesting that each variant has a distinct transcription start site.

**Figure 1:**
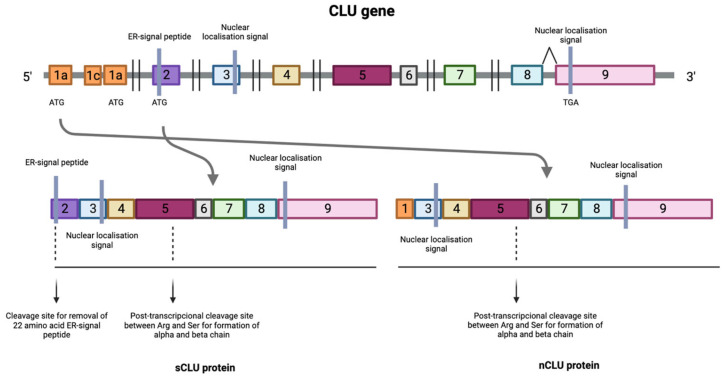
Transcription process of the CLU gene where the numbers indicate the different exons that make up the *CLU* gene. Transcription of the protein can be initiated at the mRNA synthesis initiation codon in exon 2, resulting in the formation of the mature form of the protein or secreted isoform (sCLU). In situations where this maturation pathway does not occur, a process known as alternative splicing occurs between exons 1 and 3, resulting in the deletion of exon 2 generating a nuclear and functional isoform (nCLU). Images were created using Biorender.com (accessed on 3 August 2023).

The first 22 amino acids of this sequence encode a signal that directs translocation of the preprotein to the endoplasmic reticulum (ER), where that sequence is removed and N-glycosylation of 6 asparagine residues occurs, as well as the formation of 4 or 5 disulfide bridges [8]. The protein then translocates to the Golgi apparatus, where it undergoes a more complex glycosylation that promotes its maturation, reaching a weight of 70 to 80 kDa. Subsequently, the highly glycosylated protein is cleaved between residues 227 and 228 and bound by a disulfide bond, leading to the formation of the mature form of the protein or secreted isoform (sCLU). This isoform can be internalized by other cells and stabilize the synthesis of the p53 protein, which is involved in the activation of cell cycle arresting genes, thus can inhibit cell proliferation [9,10] (Figure 1).

In situations where this maturation pathway does not occur, a process known as alternative splicing occurs between exons 1 and 3, resulting in the deletion of exon 2. This produces a protein that lacks the ER translocation signal, generating a non-functional 49 kDa prenuclear isoform (pnCLU). However, under stress, this isoform transforms into a nuclear and functional 55 kDa isoform (nCLU) [8]. The nuclear isoform translocates to the nucleus and, through its binding to the Ku70 protein, promotes cell apoptosis [11,12,13,14] (Figure 1).

On the other hand, there may also be a nearly mature, presecreted 53 KDa isoform (psCLU), which binds to the GRP78 (Bip) chaperone in the ER under stress and translocates to the mitochondrion. There, it binds to the activated form of the Bax protein, which modulates its ability to form homodimers and, in turn, inhibits the formation of Bax-Bak complexes [15]. These complexes, if not inhibited, would induce the apoptotic signaling cascade, demonstrating that clusterin has antiapoptotic activity. Furthermore, when the psCLU isoform is found in the cytoplasm, it can stabilize the Ku70-Bax complex, thus preventing the arrival of Bax into the mitochondria [9]. In the cytoplasm, it can also bind to cytotoxic substances and promote their proteasomal degradation, which further contributes to its antiapoptotic activity [16].

CLU expression can be induced by various factors, such as oxidative stress, chemotherapeutic agents, ionizing and ultraviolet radiation, estrogen, or androgen deprivation in hormone-sensitive tumors, or her2-neu receptor blockade [17,18]. In response to proapoptotic stimuli of various origins, CLU expression increases to protect and enable cell survival. There are two forms of CLU with opposite functions: the sCLU secretory form has a cytoprotective role, whereas the nCLU form can promote cell death [19,20]. Each form of CLU may be subject to its own regulatory pathway.

The regulation of CLU depends not only on the form of the protein, but also on the cell type. In addition to being found in Sertoli cells [21], CLU can be found in a wide variety of cells, such as motor neurons [22], dermal fibroblasts [23] and epithelial cells [24]. CLU expression in these varies considerably and this is thought to be due to both tissue type and different regulatory pathways acting in physiological or pathological situations [2,25]. Various factors, such as TGF-β, NGF (Nerve Growth Factor), EGF (epidermal growth factor), cytokines such as TNF-α and IL-1, IL-2, and IL-6, are involved in the regulation of CLU synthesis [2]. The Wnt signaling pathway and transcription factors such as TCF1, Jak/STAT1 (Signal Transducers and Activators of Transcription 1) and IGF1 (Insulin-Like Growth Factor 1) also play a role in CLU expression [26,27]. In addition, ATM (Ataxia Telangiectasia-Mutated) has been found to act as a sensor of DNA damage, being necessary for the induction of the IGF1-sCLU axis after ionizing radiation [28]. On the other hand, Ha-Ras and c-myc oncogenes can downregulate CLU expression [10]. CLU regulation is mediated by several elements present in the CLU promoter region, such as AP-1 (Activator Protein-1), AP-2 (Activator Protein-2), SP-1 (Stimulatory Element-1), CLE (Clusterin Element), heat shock element (HSE)-like sequences, TCF (T-Cell Factor), NF-κB (Nuclear Factor Kappa-Light-Chain-Enhancer of Activated B Cells) and STAT1 binding sites [29,30]. CLU can also be epigenetically regulated by DNA methylation and histone acetylation due to the presence of CpG island-rich domains [31,32].

### Clusterin and Its Implication in Cancer

Clusterin has been the subject of research in relation to cancer due to its participation in different aspects of the development and progression of this disease. This protein, which acts as a chaperone and whose production is stimulated by stressful situations, regulates its expression by various factors operating both inside and outside the cells. These factors include lack of growth factors, activation of the NF-kB pathway, exposure to radiation or chemotherapeutic treatments, among others [6].

Clusterin expression also appears to be influenced by epigenetic changes and related processes, such as DNA methylation or histone modification. The clusterin promoter region contains multiple CpG sites susceptible to methylation [33]. In addition, histone modifications, such as trimethylation of lysine 9 of histone 3 (H3K9me3) and trimethylation of lysine 4 of histone 3 (H3K4me3), play a predominant role in clusterin regulation in colon cancer cells [32].

Clusterin levels may vary in different types of cancer and depending on the tumor type. For example, clusterin expression has been observed in lung [34], gastric [35], ovarian [36], breast [37,38], colon [39], bladder [40], prostate [41], liver [42], pancreatic [43], melanoma [44], esophageal squamous cell cancer [45], anaplastic large cell lymphomas [46], osteosarcomas [47] and prostate [23,48] (Table 1):

Furthermore, recent studies have suggested that clusterin plays a regulatory role in tumorigenesis by influencing cancer stem cell (CSC) survival, promoting tumor growth, and contributing to epithelial–mesenchymal transition (EMT) and the acquisition of resistance to chemotherapeutic treatments when cells are faced with stressful situations [49]. Positive upregulation of Sec62 protein has also been observed to be associated with chemotherapy resistance and unfavorable prognosis in patients with rectal colon cancer [50].

As shown in Table 1, this suggests that clusterin may have both a cancer promoter and suppressor role, depending on the tumor type and specific context. When it acts as a promoter of tumor progression, it may contribute to cancer invasion and metastasis. On the other hand, in certain cases, it may have tumor suppressive effects, such as induction of apoptosis in cancer cells, inhibition of angiogenesis or promotion of anticancer immune responses.

## 2. Objectives and Methods

The aim of this paper is to review the current genetic knowledge on the role of CLU in tumorigenesis and cancer progression in general, and to discuss its possible use as a therapeutic target in colorectal cancer. For this purpose, a literature search was carried out in the MEDLINE, Embase, PubMed and Scopus databases. We also performed manual searches of study reference lists and searches of Internet resources combining several search terms such as clusterin, colorectal cancer, biomarker, tumorigenesis, targeted treatment, and personalized medicine. The language was not limited, and the years of publication were stipulated to be between 1995 and 2023. The last search was performed on 16 September 2023.

## 3. Colorectal Cancer

Colorectal cancer (CRC) is the third most frequently diagnosed cancer (10%) in both sexes, behind only breast cancer and lung cancer [51]. It ranks second in terms of overall mortality, with a 65% survival rate [52,53]. Unfortunately, approximately 25% of patients present late for consultations, leading to diagnosis at advanced or metastatic stages and, therefore, to delayed treatment [54,55]. In 2019 alone, 60% of newly diagnosed cases were advanced disease, of which 22% had distant metastases [56].

**Figure 2:**
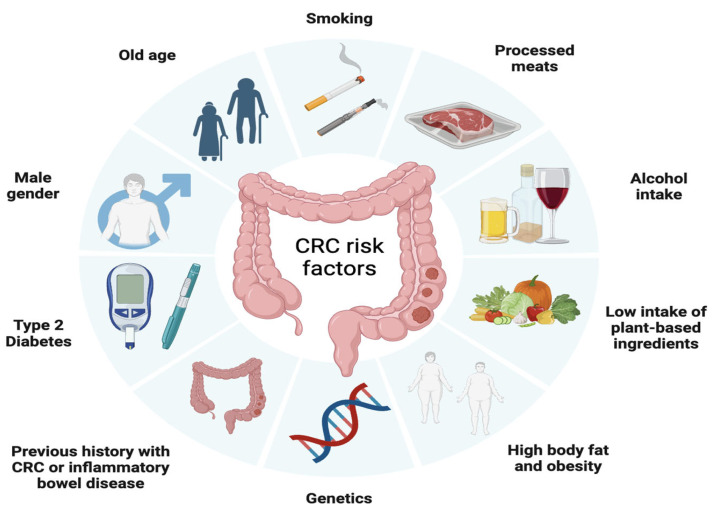
Factors associated with the development of colorectal cancer including modifiable factors (moderate to heavy alcohol consumption, smoking, a diet high in fat and low in vegetables, obesity, and sedentary lifestyle), non-modifiable factors (age, ethnicity, inflammatory bowel disease) and hereditary component. Images were created using Biorender.com (accessed on 3 August 2023).

Similar to many other types of cancer, CRC risk is influenced by a range of health behaviors and lifestyle factors such as moderate to heavy alcohol consumption, smoking, a diet high in fat and low in vegetables, obesity, and sedentary lifestyle and non-modifiable factors such as age, ethnicity, and genetic predisposition [57]. In fact, it is estimated that approximately 15% to 30% of CRC cases have a hereditary component in first- and second-degree relatives [58], with a higher risk observed in individuals who have first- and second-degree relatives affected by CRC. In addition, inflammatory bowel diseases, such as Crohn’s disease and ulcerative colitis, increase the risk of developing CRC, especially when the inflammation is chronic and long-lasting [59] (Figure 2).

The progression of colorectal cancer follows a multi-step process, starting from normal epithelial cells and advancing through distinct stages, from the formation of a premalignant lesion, commonly known as an adenoma, into a malignant lesion referred to as carcinoma. Carcinomas are characterized by invasive growth into surrounding tissues and eventually, if left unchecked, can spread systemically.

Advancements in understanding the molecular genetics and epigenetics of colorectal cancer has led to the identification of well-established alterations related to CRC such as chromosomal instability, microsatellite instability (MSI-H) and the CpG island methylator (CGI) phenotype stand out [60].

Chromosomal instability, present in 84% of cases, is related to the activation of oncogenes such as PIK3CA, K-RAS, APC, SMAD4 and TP53 [61]. Mutations in the APC oncogene occur in 80% of CRC cases [62] and lead to WNT signaling pathway activation, which promotes cells proliferation and differentiation [63]. While transformation from adenoma to carcinoma is usually caused by mutations in TP53, K-RAS and DCC 18q genes [64], progression to metastasis is normally associated with the accumulation of genetic changes in APC-KRAS-TP53 (Adenomatous Polyposis Coli–Kirsten rat sarcoma viral oncogene- the tumor suppressor p53), according to the Vogelstein model [65]. In addition, approximately 10% of CRC cases are caused by serrated neoplasia, characterized by mutations in the BRAF and K-RAS genes, which activates the MAPKinase signaling pathway, which has a main role in the regulation of gene expression, cellular growth, and survival [62,66,67].

Microsatellite instability (MSI-H) is present in 15% to 20% of CRC cases and is due to hypermethylation of the hMSH2 and hMLH1 promoters [68], as well as to mutations in mismatch repair (MMR) genes. On the other hand, the CpG-rich island (CGI) methylating phenotype involves methylation of the 5’ ends of half of all genes with short, CpG dinucleotide-rich sequences [69].

**Figure 3:**
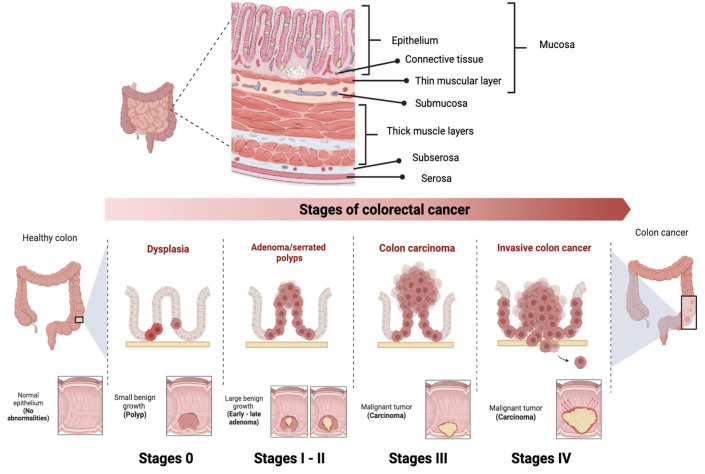
Stages of colorectal cancer depending on the extent of the tumor and the presence of metastases. The TNM staging system is used to evaluate cancer and determine its stage. It focuses on Tumor (T), describing the depth of growth of the primary tumor into the intestinal lining, with categories ranging from T0 (no cancer) to T4b (invasion of other organs or structures). The results of T, along with the assessment of Lymph Nodes (N) and Metastases (M), are combined to assign a stage to the cancer, ranging from 0 to IV. Images were created using Biorender.com (accessed on 3 August 2023).

CRC is classified into different stages depending on the extent of the tumor and the presence of metastases (Figure 3) [70]. Stage 0 (carcinoma in situ) implies that the cancer cells are found only in the innermost layer of the lining of the colon or rectum, without invading nearby tissues or spreading to lymph nodes or other parts of the body. In stage I, cancer has grown through the innermost layer of the lining, but has not spread to lymph nodes or distant organs. In stage II, cancer has grown through the lining of the colon or rectum, but has not spread to lymph nodes or distant organs. In stage III, the cancer has invaded nearby lymph nodes, but has not reached distant organs. In stage IV, the cancer has spread to distant organs. Prognosis and treatment vary at each stage, and may include surgery, radiation therapy, chemotherapy, and targeted therapy or immunotherapy [71].

Since the 5-year survival rate is 91% for stage I, decreasing to 72% for locally advanced stage disease and dropping further to 14% for stage IV [56], there is a current need for the improvement of CRC prevention and screening programs that allow for early detection. Nowadays, colonoscopy is the first option for the patient at is at higher risk of CRC due to genetic syndromes, personal or family history of colorectal cancer or the presence of precancerous polyps. Despite being a relatively expensive invasive procedure, with limitations due to the intestinal preparation required and the complications of the procedure itself [72], colonoscopy represent the most decisive test for detecting CRC because it allows the entire colon to be analyzed, biopsies of suspicious lesions to be obtained and the polyp to be removed in the same session. with a 5% miss rate.

If the patient is not at high risk, the first option is the test based on the detection of blood in stool. Despite its low sensitivity, this test was the first one used for population screening because it is the most economical and the least invasive so far available, reason why it is currently being replaced by methods such as the fecal immunohistochemical test [72]. This test was firstly approved in 2014 by the US Food and Drug Administration (FDA) and recommended for screening tests in asymptomatic patients aged 50–85 years [73].

Conventional treatments used to treat CRC include surgery, chemotherapy, and radiotherapy, alone or in combination, depending on the location [71]:

- *Surgery*

Total excision through surgery is the option used to treat localized CRC if the tumor location is easily accessible [74]. Since complete elimination of all cancer cells is not always possible so approximately 66% of stage II and III CRC patients must undergo additional treatments, where adjuvant chemotherapy and/or radiotherapy are included, respectively [75] and, in addition, 54% of patients often relapse even after undergoing adjuvant treatment [76]. This evidences the need for alternative treatments and more effects to treat CRC patients [74].

- *Radiotherapy*

Neoadjuvant radiation therapy is another important clinical option available to treat CRC, especially for those at an intermediate or advanced stage for whom surgery is not feasible or chemotherapy cannot be tolerated well. However, the use of radiotherapy is quite limited as it has low sensitivity for CRC and high toxicity in surrounding healthy tissues [77]. This neoadjuvant treatment can be given as a short course followed by surgery or as chemoradiotherapy with 5-fluorouracil or capecitabine, where possible [78].

- *Chemotherapy*

The first chemotherapeutic used against CRC was 5-fluorouracil (5-FU) [79]. Its combination with leucovorin became the standard for metastatic colon and rectal cancer (mCRCC) allowing a median overall survival of 8 to 9 months. Years later, the approval of oxaliplatin and the demonstration that it induces cell death by immunogenic mechanisms, allowed the establishment of the current standard chemotherapy of 5-FU and oxilaplatin, commonly known as FOLFOX, which has demonstrated a has superior efficacy with a median survival of 18 to 20 months [80,81].

Recently, the Chinese Clinical Oncological Society has recommended the application of neoadjuvant chemoradiotherapy in patients with CRC, specifically with sigmoid colon cancer, and with a locally advanced stage (T4b) since the response of such treatment can be increased from 26.3% to 38.1%. However, whether MSI-H patients can benefit from this approach, remains as a controversial issue [82]. Postoperative adjuvant chemotherapy is recommended for all stage III CRC [83].

- *Targeted treatments*

### 3.1. Monoclonal Antibodies

After 20 years of translational and clinical research, the epidermal growth factor receptor (EGFR) family and its intracellular signaling pathways constitute one of the foundations of molecular targeted therapy for CRC [84]. EGFR serve as cell-surface protein receptors for the peptide ligands of the epidermal growth factor (EGF) family with enzymatic tyrosine kinase activity [84]. Ligand binding induces receptor conformational change, activation, and subsequent phosphorylation of intracellular tyrosine residues, which leads to the activation of different intracellular pathways including the RAS-RAF-MEK-MAPK and PTEN-I3K-AKT-mTOR pathways, which play a main role the regulation of cell proliferation, survival, dissemination, and angiogenesis [85]. Given the role of these processes in cancer development and tumor progression, targeting the EGFR has become a key strategy in the treatment of metastatic colorectal cancer (mCRC) [86,87].

For its part, the RAS family comprises four genes (*KRAS4A*, *KRAS4B*, *HRAS* and *NRAS*) which are among the most frequently altered oncogenes in human cancer, encode for four proteins with pivotal roles in cells signaling. KRAS4A (K-RAS), the predominant splice variant, and part of the RAS/MAPK pathway. Upon stimulation by upstream receptors such as EGFR, KRAS switches from inactive to active state, and promotes RAF recruitment to the cell membrane, leading to RAF dimerization and activation of downstream effector pathways. *KRAS* mutations, which are present in approximately 40% of CRC cases, determine constitutive activation of RAS, and promote tumorigenesis as well as modulation of the tumor microenvironment by inducing immune escape and cancer progression [88], reason why RAS mutations are generally associated with poor prognosis and low response to conventional CRC therapies [89]. On the other hand, BRAF mutations, which are seen in 10% to 15% of CRC cases [90], usually lead to constitutive activation of the mitogen-activated protein kinase (MAPK) signaling pathway, conferring high clinical aggressiveness, resistance to anti-EGFR monoclonal antibody therapy, and poor survival [91].

The first successful step towards personalized cancer medicine has been the definition of different treatment options and sequences, which are based on tumor molecular stratification [92]. Since the validation of *KRAS* and *BRAF* mutations as predictive biomarkers to anti-EGFR monoclonal antibodies in mCRC, regulatory agencies, such as the FDA and the European Medicines Agency (EMA), established the need to assess K-RAS and BRAF mutation status for patient stratification and management.

Targeted therapies based on monoclonal antibodies has become a pivotal therapeutic strategy to treat many solid tumors (Table 2).

Bevacizumab (Avastin; Genentech, South San Francisco, CA, USA), a human antiendothelial growth factor receptor 2 (VEGF) monoclonal antibody, anti-VEGF-A, was the first antigenic drug to be successfully added to the therapeutic armamentarium for CRC. Given its very modest action as a single agent the FDA approves its use in combination with fluoropyrimidine [93]. Nowadays, bevacizumab is widely used for the treatment of patients with mCRC in combination with oxaliplatin-based chemotherapy, due to evidence of improved patient survival [94], either as first-line or second-line therapeutic options [95,96].

Other available drugs approved as second-line treatment for mCRC, Ziv-aflibercept (Zaltrap; Regeneron Pharmaceuticals, Tarrytown, NY, USA) a fully humanized recombinant fusion protein that blocks VEGF-A with higher binding affinity than Bevacizumad [97] and Ramucirumab (Cyramza; Eli Lilly, Indianapolis, IN, USA) a fully humanized IgG1completely humanized monoclonal antibody (mAb) targeting the extracellular domain of VEGF, both of which have demonstrated efficacy for second-line use against CRC in combination with leucovorin and ironotecan (FOLFIRI) or irinotecan alone.

Monoclonal antibodies against the epithelial growth factor receptor (EGFR) include the murine human IgG1 mAB Cetuximab (Erbitux; Bristol-Myers Squibb, New York, NY, USA), which is a murine human IgG1 mAB, and the human IgG2 mAb Panitumubab (Vectibix; Amgen, South San Francisco, CA, USA), both of which bind to the extracellular domain of EGFR, resulting in the negative regulation of prooncogenic signaling pathway. Cetuximab, binding to natural killer cells can also promote the immune-mediated antitumor response leading to antibody-dependent cell-mediated cytotoxicity. This is not the case with Cetuximab, where antibody-dependent cell-mediated cytotoxicity is not activated [98,99]. Cetuximab or Panitumumab in combination with chemotherapy may increase the survival rate over chemotherapy alone. However, these are used in RAS WT tumors and not in tumors with K-RAS mutations because EFGR inhibition only has efficacy in CRCs where the intracellular signaling machinery is composed of normal and non-mutated proteins [100]. That is, its use is restricted to selected CRC patients where targeting is based on molecular biomarkers predictive of non-response, i.e., activating RAS BRAF mutations [101]. For 5–10% of selected patients, combination therapy increased overall survival [102].

Currently, encorafenib (Braftovi; Pierre Fabre, Paris, France), a BRAF inhibitor, whose combination with Cetuximab is showing promising results in clinical trials [103].

### 3.2. Immune Checkpoint Inhibitors

Immune checkpoint inhibitors are a new alternative in cancer treatment as they use the patient’s own immune system to fight cancer cells by specifically targeting the antigens of the malignant cells, alerting the immune system to their presence, and attempting to eradicate the cancer through the immune response [104]. PD-1 is a transmembrane protein that is expressed on the cell surface and overexpressed in inflammatory environments or on the surface of tumor cells [105] to which its ligand (PD-1L) binds causing inhibition of tumor cell apoptosis and transformation of effector T cells into regulatory T cells, this is, T cell anergy [106]. CTLA-4 is responsible for modulating T-cell activation and has therefore been targeted therapeutically within the immune checkpoint. By binding to B7-1 (CD80) and B7-2 (CD86) ligand on antigen-presenting cells, it regulates the activation of tumor-reactive T cells [100].

The specificity of this immunotherapy renders normal cells, devoid of the cancer antigens, unaffected. In this regard, the results have been very promising, as those patients who responded well to this immunotherapy have a better prognosis and a better quality of life [74].

Patients harboring MSI-H/dMMR type tumors represent a population of mCRC patients who, at present, appear to benefit most from these immune therapies as they have many somatic mutations that produce multiple neoantigens that can be recognized as foreign [107,108]. However, they account for only 5% of mCRCs so greater efforts are needed to apply this immunotherapy in MSS/MMR patients [104].

Therefore, immune checkpoint-based immunotherapy includes programmed cell death protein 1 (PD-1) inhibitors and anticytotoxic T-lymphocyte antigen 4 (CTLA-4) antibodies. In this regard, there are three FDA-approved drugs to treat MSI-H/MMR patients with mCRC: two against PD-1 and one against CTLA-4.

In June 2022, Pembrolizumab (Keytruda; Merck, Kenilworth, NJ, USA), IgG4 mAbs against PD-1, was approved to treat MSI-H/MMR patients. Results showed that patients taking it have a progression-free survival rate to metastasis of up to 78% [109] (Table 1). However, patients have reported adverse reactions in 20% of cases such as decreased appetite, nausea, rash, dyspnea, or diarrhea, among others.

Another approved PD-1 inhibitor is Nivolumab (Opdivo; Bristol-Myers Squibb, New York, NY, USA) which has shown durable responses and 12-month overall survival in 69% of patients with mCRC [110]. The combination of Nivolumab with Ipilimubab (Yervoy; Bristol-Myers Squibb, New York, NY, USA) drug directed against CTLA4, shows a response rate of up to 94% which has suggested that combination checkpoint therapy may improve efficacy in MSI-H/dMMR patients (Table 2) [111].

While clearly MSI-H/MMR patients are benefiting the most from these therapies, important steps are being taken within clinical trials to help all CRC patients [112].

## 4. Clusterin as a Therapeutic Target in Colorectal Cancer

In recent years, the relationship between clusterin and colorectal cancer (CRC) has been investigated from a genetic perspective. Numerous studies have focused on examining both the expression and function of clusterin in the context of CRC, as well as genetic alterations associated with this protein in relation to the disease.

In terms of immunohistochemistry, patterns of clusterin expression and its functional implications in CRC have been identified. A study conducted by Pucci et al. observed the presence of clusterin in highly aggressive colorectal tumors and metastatic nodules. Furthermore, it was noted that the localization of clusterin within cells varied from the nucleus in healthy tissues to the cytoplasm in malignant tumors, which has been linked to the disease’s status. This suggests that clusterin plays a protective role against apoptosis in situations of physiological stress, contributing to the acquisition of aggressive behavior in cancer, depending on the isoform expressed at that time [11].

Another study by Bertuzzi et al. suggested clusterin as a potential biomarker, as an increase in circulating clusterin was found in pre-diagnosis samples. This could improve the identification of individuals at risk of colorectal cancer and the planning of prevention strategies [113].

In the realm of transcriptomic and proteomic analysis of clusterin expression, the work of Artemaki et al. employed clusterin mRNA as a novel biomarker for predicting colorectal cancer. They found that elevated levels of clusterin mRNA in colorectal cancer tumors could predict an adverse prognosis for patients with Grade II and/or TNM stage III cancer [114]. In contrast, the study by Kopylov et al. found clusterin present in early stages of the disease; however, their study was limited by the small size of cohorts and the lack of clinical stratification [115].

Pucci et al. also demonstrated that the nuclear form of clusterin was present in healthy mucosal cells and played a role in regulating the cell cycle and apoptosis. This suggests that under normal conditions, clusterin induces apoptosis and regulates the cell cycle. However, in the case of cell irradiation, nuclear clusterin might interfere with DNA repair, potentially leading to cell death [116]. Furthermore, subsequent studies by Komuro et al. observed that the levels of the proteins Ku70 and Ku80 were related to tumor sensitivity to radiotherapy and response to chemotherapy in human colorectal cancer [117,118].

Building upon these findings, Mazzarelli et al. conducted a study to assess the expression of the Ku70/80 heterodimer in human colorectal carcinogenesis, from adenoma to carcinoma. They found that upregulation of the non-homologous end joining (NHEJ) system in adenomas and colon carcinomas could be involved in increased genomic rearrangements and chromosomal abnormalities, potentially contributing to tumor progression. This information is crucial for understanding DNA repair mechanisms and improving the treatment of colorectal cancer patients [119].

Pucci et al. carried out both in vivo and in vitro studies to investigate the relationship between Ku70-Ku80-sClu as a prognostic marker and its implication in treatment response. They proposed Ku70-80-sClu as a molecular cluster predictor of neoadjuvant chemoradiotherapy response in locally advanced rectal cancer patients, although further studies in larger cohorts are needed to confirm these findings [120].

Additionally, in vitro studies have been conducted to examine clusterin expression in response to various chemotherapeutic agents and its relationship with the heterogeneity of cancer stem cell populations. Kevans et al. used the SW480 cell line and found that clusterin overexpression, particularly under hypoxic conditions, was associated with increased sensitivity of colorectal cancer cells to FOLFOX treatment. These findings suggest that in vivo tumors expressing high levels of clusterin, especially under hypoxic conditions, may benefit from treatments such as FOLFOX [121]. Engel et al. used patient-derived colorectal cancer organoids to study the heterogeneity of stem cell populations represented in primary tumors. They found that clusterin was enriched in organoids resistant to 5-FU, correlating with reduced survival and increased disease recurrence in patients [122].

Grosgeorges et al. employed clusterin, along with other markers (B2M and TIMP-1), in the detection of metastatic colorectal cancer (mCRC) using circulating cell-free mRNA from plasma samples. They observed that the concentration of these mRNAs was significantly higher in plasma samples from mCRC patients compared to healthy individuals. This could have implications for the diagnosis and treatment of colorectal cancer [123]. In addition, Insua et al. used circulating tumor cells (CTCs) in patients with metastatic colorectal cancer to measure the levels of mRNA from a panel of six genes (*GAPDH*, *CLU, VIL1*, *TIMP1*, *LOXL3*, and *ZEB2*), where they identified that this approach effectively allowed the identification of patients who did not respond to first-line chemotherapy and had worse progression-free survival and overall survival rates [124,125].

Recently, Du and colleagues have employed machine learning algorithms to develop a risk prediction model for CRC, enabling precise patient stratification and the identification of genes associated with disease prognosis. They used CRC data extracted from the Gene Expression Omnibus (GEO) databases GSE126092 and GSE156355, as well as datasets from The Cancer Genome Atlas (TCGA), to conduct bioinformatics analyses that identified differentially expressed genes (DEGs) [126]. The CRC risk prediction model was based on a combination of genes, including CHGA, CLU, PLK1, AXIN2, NR3C2, IL17RB, GCG, and AJUBA. It was determined that CLU, PLK1, and IL17RB are genes that can be considered prognostic factors in CRC. Moreover, this model not only offers improved patient stratification and treatment guidance but also provides a deeper biological insight into understanding survival conditions in CRC [126].

## 5. Conclusions

Although the treatment of colorectal cancer has been evolving in recent years, the mortality rate of the disease is still quite high due, among other things, to the delay in the diagnosis of the disease and the acquisition of resistance to available treatments. This has generated the need to identify and develop new therapeutic targets that allow, in addition to improving mortality figures, to understand the mechanisms of resistance to existing treatments to overcome them. Colorectal cancer is a heterogeneous process that usually occurs in several steps, so it is not surprising that there are many possible prognostic biomarkers of the disease. Throughout the review, we have seen the importance of the clusterin protein in tumorigenesis and cancer prognosis. In this sense, the protein and its mRNA have been studied as biomarkers in different types of cancer including lung, ovarian, breast, bladder, or hepatocellular cancer, but the literatus is scarce with respect to colorectal cancer where some studies point to its potential as a biomarker in the prognosis and prevention of CRC in early stages. These findings underscore the importance of continuing research in this field as it is used to predict the success of neoadjuvant treatments in CRC.

## Figures and Tables

**Table 1:** Relevant results in studies related to clusterin in different types of cancer.

Cancer Type	Results
Lung	More than 80% of non-small-cell lung cancers are immunoreactive for clusterin [34].
	Clustering silencing using antisense oligonucleotides (ASOs), or short interfering double-stranded RNAs (siRNAs) significantly improve sensitivity to paclitaxel in vivo [34].
Gastric	sCLU overexpression is involved in the progression of human gastric carcinomas and it seems that its oncogenic function could be associated with p53 disfunction [35].
Ovary	Clusterin overexpression is more frequently detected in metastatic lesions more frequently than in primary tumors [36].
	Cytoplasmatic clusterin overexpression in ovary carcinomas is inversely correlated with tumor apoptotic index [36].
Breast	Unlike benign lesions, atypical hyperplasias, intraductal carcinomas and invasive carcinomas are characterize by clusterin overexpression [37].Clusterin expression tended to be inversely correlated with the apoptotic rate, indicating that gene expression is not required for apoptotic cell death. This suggests that CLU may play a role in breast carcinogenesis [37,38].
Colon	sCLU is overexpressed while nCLU is downregulated [39].
	Clusterin expression may help identify patients with more aggressive tumors who may benefit from targeted therapies [40].
Bladder	The recurrence-free survival rate of patients with strong clusterin expression is significantly lower than in patients with weak expression [41].
Hepatic	*CLU* expression in peripheral blood mononuclear cells (PBMC) has been proposed as a prospective screening marker for hepatocellular carcinoma along with other genes [42].
Pancreas	Clusterin is highly expressed in stages I and II (well-differentiated and moderately differentiated cancers) [43] and it expression is not significantly associated with apoptosis [43].
	Clusterin-positive patients with pancreatic cancer survived significantly longer [43].
Melanoma	Clusterin overexpression is associated with increased drug resistance and increased survival of tumor cells [44], and its silencing reduces drug resistance and survival of melanoma cells both in vitro and in vivo [44]. Mice pretreatment with antisense oligonucleotide targeting CLU is associated with a significantly improved tumor response to dacarbazine compared to control [44].
Esophageal squamous cells	High stromal CLU expression is associated with poor locoregional, overall and distant progression-free survival [45].
	Esophageal Squamous Cancer Cell patients with high CLU expression in both epithelium and stroma have the shortest survival time [45].
Anaplastic large cell lymphomas	Clusterin expression is not related to anaplastic lymphoma kinase-1 expression and in reactive lymphoid tissues, only fibroblastic reticular and follicular dendritic cells show positive expression by inmunohistochemistry [46].
	Although CLU role in anaplastic large cell lymphoma (ALCL) is unknown, the unique expression of clusterin within this category of lymphoma provides an additional marker for the diagnosis of ALCL [46].
Osteosarcomas	sCLU is expressed in osteosarcoma and its overexpression is associated with metastasis and chemoresistance [47].
	Silencing sCLU inhibits metastasis and improves chemosensitivity in osteosarcoma cells [47].
Prostate	A significant decrease in clusterin expression compared to corresponding benign tissues has been observed [23] and reduction in hormone-resistant prostate carcinomas [48].

**Table 2:** Types of immunotherapeutics used to treat colorectal cancer.

Type	Molecular Target	Immunotherapeutics	Type of Patient
Monoclonal antibodies	VEGF-A	Bevacizumab	CRCm
	VEGF-A	Ziv-aflibercept	
	Extracellular domain of VEGF	Ramucirumab	
	Extracellular domain of EGFR	Cetuximab	CRCm with RAS WT tumors
		Panitumumad	
	BRAF	Encorafenib	CRCm with RAS WT tumors
Immune checkpoint inhibitors	PD-1	Pembrolizumab	MSI-H/dMMR
	PD-1	Nivolumab	CRCm
	CTLA4	Ipilimubab	MSI-H/dMMR
